# Thromboelastography-Based Risk-Stratified Transfusion Strategy in Acute Stanford Type A Aortic Dissection: A Predictive Model and Prospective Validation

**DOI:** 10.3390/jcm15093446

**Published:** 2026-04-30

**Authors:** Jiawei Zhu, Qiuyong Guo, Yi Jiang, Xinlong Tang, Xiyu Zhu, Hoshun Chong, Yunxing Xue, Jun Pan, Jinfeng Yu, Qing Chen, Fudong Fan, Dongjin Wang

**Affiliations:** 1 Department of Thoracic and Cardiovascular Surgery, Nanjing Drum Tower Hospital Clinical College of Nanjing Medical University, Nanjing 210008, China; njglyycardiaczjw@163.com (J.Z.); jstangxinlong@njglyy.com (X.T.); albert_xue@163.com (Y.X.); pj791028@njglyy.com (J.P.); 2 Institute of Cardiothoracic Vascular Disease, Nanjing University, Nanjing 210008, China; s2023039003@pumc.edu.cn (Q.G.); drjiangy@pumc.edu.cn (Y.J.); zhuxy_nju@163.com (X.Z.); hoshunchong@163.com (H.C.); 3 Department of Thoracic and Cardiovascular Surgery, Nanjing Drum Tower Hospital, Chinese Academy of Medical Science & Peking Union Medical College, Peking Union Medical College Graduate School, Nanjing 210008, China; 4 Department of Thoracic and Cardiovascular Surgery, Nanjing Drum Tower Hospital, The Affiliated Hospital of Nanjing University Medical School, Nanjing 210008, China; 5 Department of Transfusion Medicine, Nanjing Drum Tower Hospital, The Affiliated Hospital of Nanjing University Medical School, Nanjing 210008, China; lyxx201707@163.com (J.Y.); qchen@njglyy.com (Q.C.)

**Keywords:** thromboelastography, risk-stratified transfusion protocol, acute stanford type A aortic dissection, perioperative excessive bleeding, predictive model

## Abstract

**Objectives**: Perioperative blood transfusion for acute type A aortic dissection (ATAAD) lacks clinical guidelines. This study aims to investigate the application of a thromboelastography (TEG)-based risk-stratified transfusion protocol in these patients. **Methods**: We conducted a two-stage study. Firstly, a retrospective analysis of ATAAD patients undergoing surgery in 2023 was performed to identify predictors of postoperative/perioperative excessive bleeding and develop a predictive model. Subsequently, a single-center prospective validation study was conducted in 2024, comparing a TEG-based risk-stratified transfusion protocol against conventional empirical transfusion. **Results**: In the retrospective phase (*n* = 57), 18 patients (31.6%) developed perioperative excessive bleeding. Preoperative activated clotting time (ACT) and TEG parameters (K-time) were independent predictors. A predictive model incorporating these variables achieved an AUC of 0.788. In the prospective phase (*n* = 47), 21 patients received the TEG-based risk-stratified transfusion protocol. Compared to the conventional group, the TEG risk-stratified group exhibited significantly lower postoperative drainage volume (*p* = 0.046), a reduced incidence of perioperative excessive bleeding (4.8% vs. 34.6%, *p* = 0.033), and lower transfusion costs (*p* = 0.029), without an increase in total transfusion volume. **Conclusions**: Preoperative ACT and TEG parameters effectively predict perioperative excessive bleeding in ATAAD patients. Implementing a TEG-based risk-stratified transfusion protocol optimizes blood product utilization, improves clinical outcomes, and reduces costs, offering a promising evidence-based approach for perioperative management.

## 1. Introduction

Acute type A aortic dissection (ATAAD) represents one of the most lethal cardiovascular emergencies, with mortality rates increasing by 1% per hour [1]. Surgical intervention remains the most effective treatment. Nevertheless, the incidence of perioperative excessive bleeding in surgically treated patients with ATAAD remains high due to factors such as preoperative hypercoagulability [2,3], extensive surgical trauma, deep hypothermic circulatory arrest (DHCA), and prolonged cardiopulmonary bypass [4,5]. Currently, clinical practice relies on postoperative continuous chest tube drainage volume to guide transfusion decisions [6,7,8]. Current guidelines for perioperative blood management primarily adhere to the principles of preoperative hemoglobin optimization, reduction of iatrogenic blood loss, and enhancement of patient tolerance to anemia. However, as noted in the 2022 ACC/AHA Aortic Disease Guidelines [1] and recent ESC guidelines [9] on patient blood management, there is a significant lack of standardized, evidence-based perioperative blood management protocols for ATAAD, with most strategies depending on preoperative hemoglobin levels and institutional experience [10], without evidence-based guidelines.

The current clinical landscape faces a critical shortage of blood product resources [11], compounded by their inappropriate use. While emerging technologies like intraoperative autologous transfusion and platelet-rich plasma separation technology have partially alleviated perioperative blood supply constraints in cardiothoracic vascular surgeries, patients with ATAAD still inevitably require blood transfusion [12,13]. Inappropriate transfusion protocols also significantly elevate the risk of transfusion-associated complications [14]. Excessive packed red blood cell (PRBC) transfusion markedly increases postoperative deep vein thrombosis and pulmonary thromboembolism incidence [15]. In pediatric cardiac surgery, transfusion overload substantially raises pulmonary complication rates [16]. Therefore, developing optimized, evidence-based transfusion strategies is imperative.

Thromboelastography (TEG) is an advanced viscoelastic hemostatic assay that dynamically monitors whole blood coagulation status through specialized sensors. It has proven valuable in guiding transfusion in trauma, liver surgery, and cardiac surgery. Emerging evidence supports its utility in trauma resuscitation, with TEG-based transfusion strategies demonstrating improved outcomes [16]. Emani recently demonstrated that TEG-based transfusion protocols significantly reduce postoperative bleeding complications in pediatric cardiac surgery [17]. In surgical practice, TEG-based transfusion management has effectively reduced perioperative blood product utilization in procedures such as hepatic and obstetric surgeries, without increasing postoperative complication rates [18,19,20]. However, its application specifically in ATAAD perioperative transfusion management remains unexplored.

This study aimed to develop and validate a TEG-based risk-stratified transfusion protocol for patients with ATAAD during the perioperative period. We conducted a single-center, prospective, proof-of-concept study with a pilot design to evaluate this TEG-based strategy. We hypothesized that the TEG-based risk-stratified transfusion protocol would effectively reduce the incidence of perioperative excessive bleeding and subsequently improve clinical outcomes in these patients.

## 2. Materials and Methods

### 2.1. Subject

This two-phase study was conducted from 1 January 2023 to 31 December 2024. The retrospective component of this study identified predictors of perioperative excessive bleeding in ATAAD patients and developed a predictive model, which informed the design of a TEG-based risk-stratified perioperative transfusion protocol. The prospective component of this study evaluated this strategy in a single-center exploratory trial.

The study was approved by the Institutional Review Board of The Affiliated Hospital of Nanjing University Medical School (Retrospective: IRB 2022-157-01; Prospective: IRB 2022-235-02) and conducted in accordance with the Declaration of Helsinki. Written informed consent was obtained from all participants.

This study was registered in the Chinese Clinical Trial Registry. The retrospective part of the study was registered under the registration number ChiCTR2200057197 on 3 March 2022, and the prospective part was registered under the registration number ChiCTR2200063439 on 6 September 2022. Both registrations were conducted at Nanjing Drum Tower Hospital, Affiliated Hospital of Medical School, Nanjing University.

### 2.2. Patients

**Figure 1:**
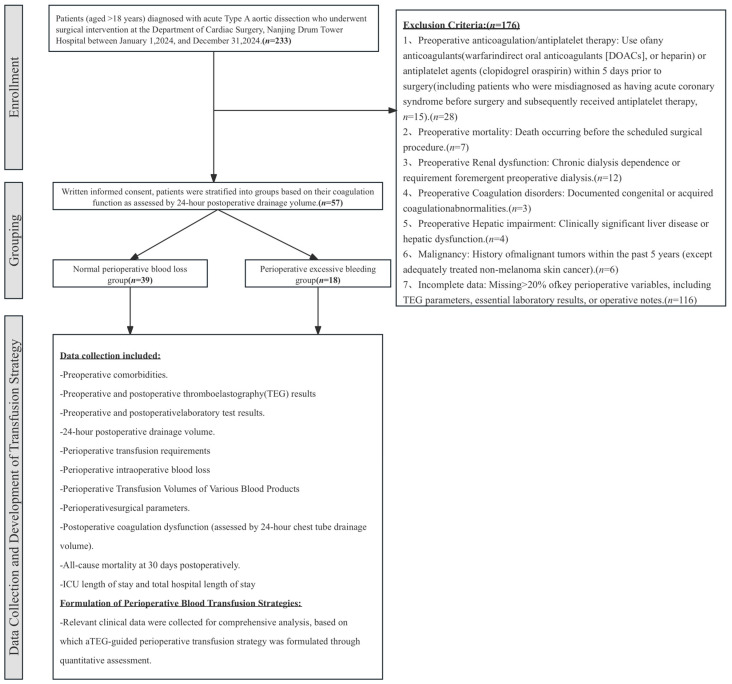
Flowchart of the Retrospective Component.

Retrospective Cohort: The retrospective component of this study included 57 patients with ATAAD who underwent surgery at the Department of Cardiac Surgery, Nanjing Drum Tower Hospital, from 1 January 2023 to 31 December 2023 [21]. We assessed perioperative excessive bleeding based on the volume of chest drainage and used it for grouping purposes (Figure 1).

**Figure 2:**
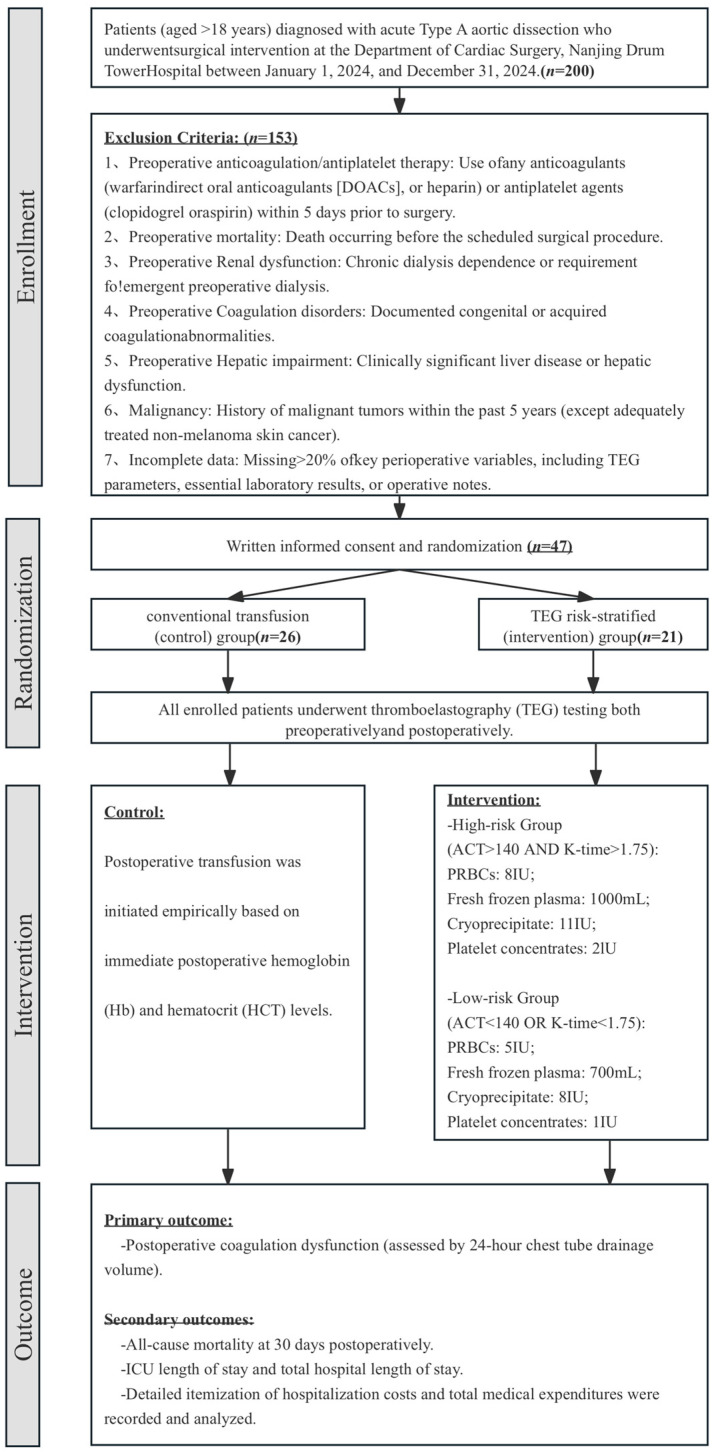
Flowchart of the Prospective Component.

Prospective cohort: This study initially intended to enroll 200 eligible patients who underwent aTAAD surgery following surgical intervention between 1 January 2024, and 31 December 2024. However, after applying the preset inclusion and exclusion criteria, a total of 153 patients were excluded. Consequently, 47 patients were prospectively included in the study. This sample size is relatively small. Therefore, this study should be considered exploratory in nature. The detailed enrollment process is shown in Figure 2. To minimize selection bias, all patients meeting the eligibility criteria were consecutively screened and enrolled without exclusion based on clinical judgment. Data were prospectively collected by research staff not involved in clinical care using standardized case report forms to reduce the risk of detection bias.

### 2.3. Randomization and Study Enrollment

Group allocation was performed by simple randomization using a computer-generated sequence created with R software (v.4.1.2). An independent statistician prepared sealed, opaque, sequentially numbered envelopes containing assignments to either the TEG-based risk-stratified transfusion group (even numbers) or conventional transfusion group (odd numbers). Envelopes were opened by treating clinicians only after eligibility confirmation (Figure 1). This was a single-blind study. Patients were blinded to their group assignment to minimize the potential influence of awareness on postoperative outcomes. However, due to the nature of the intervention, treating clinicians and outcome assessors could not be blinded to group allocation. Group allocation was performed by simple randomization using a computer-generated sequence. Envelopes were opened by treating clinicians only after eligibility confirmation and immediately prior to initiating the transfusion protocol.

For the conventional transfusion group, transfusion decisions were made by attending surgeons and intensivists based on the institutional standard practice derived from clinical experience prior to this study. This practice typically included: (1) red blood cell transfusion when hemoglobin < 8 g/dL or in the presence of active bleeding with hemodynamic instability; (2) fresh frozen plasma and platelet transfusion at the discretion of the clinical team based on evidence of microvascular bleeding and point-of-care coagulation test results. No standardized algorithm or TEG guidance was applied in this group. The explicit description of this comparator group helps reduce performance bias by ensuring transparency regarding the control intervention.

### 2.4. Definitions of Clinical Indicators and Events

Assessment of perioperative excessive bleeding: Coagulation status was evaluated based on chest tube drainage output in the perioperative period to determine the need for additional blood transfusion or reoperation [4,13].

Definition of excessive postoperative drainage: Patients meeting any of the following criteria should be diagnosed with excessive drainage: (1) Drainage volume > 500 mL within the first hour postoperatively; (2) Drainage volume > 400 mL/h for 2 consecutive hours after surgery; (3) Drainage volume > 300 mL/h for 3 consecutive hours after surgery; (4) Total drainage volume > 1000 mL within 4 h postoperatively [14].

### 2.5. TEG Protocol and Parameter Definitions

In this study, venous blood samples were collected from included patients preoperatively and postoperatively for TEG analysis. Preoperative blood specimens were obtained when the patient entered the operating room, while postoperative blood specimens were collected immediately upon the patient’s admission to the cardiac intensive care unit (CICU). All venous blood samples were promptly sent to the institutional clinical laboratory for analysis after collection. Preoperative TEG results were used for risk stratification and to guide individualized transfusion strategies after weaning from cardiopulmonary bypass (CBP), whereas postoperative TEG results were used to assess coagulation recovery and evaluate the effectiveness of the transfusion strategy, including the need for additional blood transfusion or adverse outcomes such as re-exploration for bleeding. Certified technicians performed kaolin-activated thromboelastography using a TEG^®^ 5000 Hemostasis Analyzer (Haemonetics Corporation, Boston, MA, USA) according to the manufacturer’s instructions. The following parameters were recorded: R-time, K-time, MA, α-angle, LY30, CI, EPL, G, and A.

### 2.6. Statistical Analysis

Normality of continuous variables was assessed using the Shapiro–Wilk test. Data are expressed as mean ± SD (*t*-test) or median[IQR] (Mann–Whitney U test). Categorical variables are presented as n (%) and compared with χ^2^ or Fisher’s exact test. Significant preoperative TEG parameters (*p* < 0.05) were incorporated into a predictive model. Predictive performance for hemostatic impairment was assessed by ROC analysis, with the Youden index identifying optimal cutoffs. *p* < 0.05 was considered significant.

## 3. Results

### 3.1. Characteristics of Patients in the Retrospective Study Component

During the study period (1 January 2023 to 31 December 2023), 233 patients with ATAAD were initially screened. After applying inclusion/exclusion criteria, 57 were enrolled, among whom 18 (31.6%) developed perioperative excessive bleeding. Among 233 initially screened ATAAD patients in January 2023–December 2023, 57 met the criteria for analysis. Eighteen patients (31.6%) developed perioperative excessive bleeding. The normal perioperative blood loss group had a median age of 56.00 [44.50, 72.00] years and a BMI of 25.59 [21.52, 27.64] kg/m^2^, and 69.2% were male; the perioperative excessive bleeding group had a median age of 56.50 [49.00, 61.75] years and a BMI of 24.07 [22.12, 25.50] kg/m^2^, and 88.9% were male. Intraoperative blood loss was significantly higher in the perioperative excessive bleeding group (2100.00 [1525.00, 2875.00] mL vs. 1500.00 [1200.00, 1950.00] mL, *p* = 0.012) (Table 1 and Table 2).

Preoperatively, TEG showed prolonged K-time in the perioperative excessive bleeding group (2.90 [2.15, 3.60] min vs. 1.80 [1.35, 2.50] min, *p* = 0.001). Postoperative K-time was not significantly different, but MA was significantly lower in perioperative excessive bleeding patients (50.65 [48.42, 55.20] mm vs. 58.20 [53.85, 63.65] mm, *p* = 0.001) (Table 3).

Preoperative eosinophil percentage (0.20 [0.03, 0.50] vs. 0.10 [0.00, 0.10], *p* = 0.039) and PDW (16.50 [15.93, 16.60] vs. 15.20 [12.85, 16.45], *p* = 0.027) were higher in the perioperative excessive bleeding group, while eGFR was lower (63.90 [23.70, 82.39] mL/min/1.73 m^2^ vs. 82.39 [52.05, 128.25] mL/min/1.73 m^2^, *p* = 0.028). No preoperative AST difference was observed, but postoperative AST was higher in the perioperative excessive bleeding group (43.95 [14.88, 143.28] U/L vs. 19.90 [14.10, 30.20] U/L, *p* = 0.048), as presented in Supplementary Table S1.

### 3.2. Correlation Analysis and Univariate Logistic Regression Analysis

Pearson correlation analysis of TEG parameters in patients with ATAAD revealed significant associations between preoperative and postoperative measurements. Specifically, preoperative kinetic time (K-time) demonstrated a moderate positive correlation with postoperative K-time (r = 0.60, *p* < 0.001) and a moderate negative correlation with MA (r = −0.67, *p* < 0.001). Preoperative MA showed a moderate negative correlation with postoperative K-time (r = −0.66, *p* < 0.001) and a weak positive correlation with postoperative MA (r = 0.47, *p* < 0.001). Complete correlation results are illustrated in Supplementary Figure S1.

The univariate logistic regression analysis of retrospectively enrolled patients with ATAAD is presented in Supplementary Table S2, and the TEG-related results are shown in Table 4. The results demonstrated statistically significant associations for multiple parameters, such as preoperative ACT (OR: 1.008, 95%CI 1.002–1.019, *p* = 0.048), R-time (OR: 2.146, 95%CI: 1.151–5.664, *p* = 0.049), K-time (OR: 1.848, 95%CI: 1.108–3.476, *p* = 0.034), α-Angle (OR: 0.934, 95%CI: 0.871–0.995, *p* = 0.039), and postoperative MA (OR: 0.864, 95%CI: 0.774–0.944, *p* = 0.003).

### 3.3. Development of Predictive Models and Formulation of Transfusion Strategies

**Figure 3:**
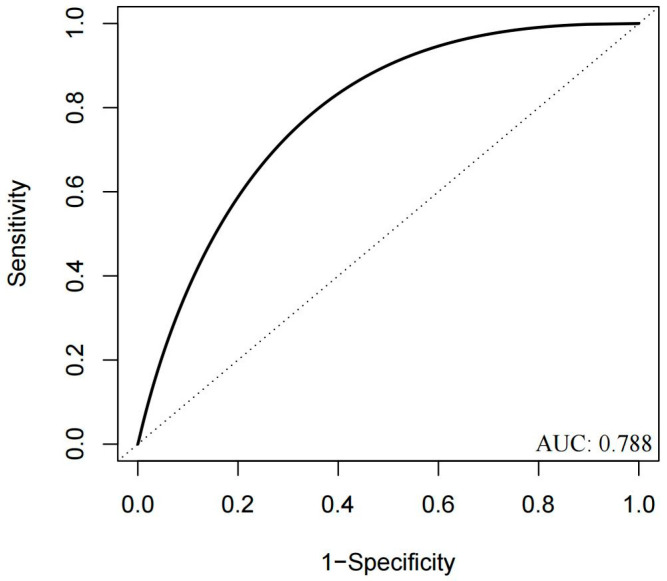
ROC curve of the predictive model. The area under the curve (AUC) was 0.788, with a 95% confidence interval (CI) of 0.655–0.901.

This study developed a predictive model for perioperative excessive bleeding using preoperative thromboelastography parameters. To identify predictors of perioperative excessive bleeding, univariate logistic analysis was first performed for all preoperative clinical variables and preoperative TEG parameters. Predictors with a *p*-value < 0.05 in the univariate analysis were entered into the final model. The variance inflation factor (VIF) was used to assess multicollinearity among the predictors, with a threshold of <5 considered acceptable. The results of the multivariate logistic regression analysis are shown in Supplementary Table S3. ROC analysis (Figure 3, Figures S2 and S3) identified optimal cutoffs at 140 s for ACT and 1.75 min for K-time via the Youden index. Based on these findings, we created a TEG-based risk-stratified transfusion protocol tailored to patients’ coagulation profiles (Table 5).

### 3.4. Characteristics of Patients in the Prospective Study Component

A novel TEG-based risk-stratified transfusion protocol for ATAAD patients was investigated in a prospective, single-center pilot study to assess its feasibility and efficacy in the perioperative period. Between 1 January and 31 December 2024, a total of 47 patients with ATAAD who were prospectively enrolled were randomized into two groups. Using a simple randomization method, 21 patients were allocated to receive a TEG-based risk-stratified transfusion protocol, while the remaining patients received conventional empirical transfusion. Due to the inherent differences in the decision-making protocols between the two transfusion strategies, blinding of the surgery surgeons to the group assignment was not feasible in this study. But throughout the trial, patients were blinded to their group assignment.

The TEG risk-stratified group showed significantly lower rates of perioperative excessive bleeding (4.8% vs. 34.6%, *p* = 0.033) compared to the conventional group. The 30-day mortality rate was 0% in the TEG risk-stratified group compared with 33.3% in the conventional transfusion group (rate difference: 33.3%, 95% CI: 0.135–0.532, *p* = 0.03). There was no significant difference in total perioperative transfusion volume between groups (*p* = 0.029), indicating that the TEG-based risk-stratified transfusion strategy improved outcomes without increasing transfusion burden (Table 6 and Table S4).

## 4. Discussion

In this study, we utilized thromboelastography (TEG) parameters to predict perioperative excessive bleeding in patients with acute type A aortic dissection (ATAAD) and implemented a TEG-based risk-stratified perioperative transfusion strategy. Through a single-center prospective study, we demonstrated that this TEG-based approach may play a significant role in reducing the incidence of perioperative excessive bleeding in patients with aTAAD compared with traditional transfusion strategies.

Current clinical evidence suggests that perioperative excessive bleeding in patients with ATAAD primarily results from coagulation dysfunction induced by prolonged cardiopulmonary bypass (CPB) [22] and a coagulation factor consumption-induced preoperative hypercoagulable state [3,23]. While activated clotting time (ACT) remains the gold standard for monitoring heparin anticoagulation during CPB [24], serving to guide heparin administration and reversal, growing evidence indicates its insufficiency as a sole indicator for comprehensive perioperative coagulation assessment. Several critical factors challenge the reliability of ACT-based testing: preoperative coagulation abnormalities [25], heparin resistance [26], individual variability in anticoagulation response and the limited sensitivity in detecting residual heparin after protamine neutralization [27,28]. The limitations of ACT testing may compromise its ability to accurately reflect postoperative coagulation changes in patients with ATAAD. To further investigate perioperative excessive bleeding, our study incorporated TEG as a complementary monitoring modality, enabling a more comprehensive assessment of the entire coagulation process.

Compared to conventional coagulation tests, TEG is a viscoelastic assay that analyzes whole blood properties to comprehensively assess hemostatic function [29,30,31]. Previous studies have demonstrated that TEG-based risk-stratified management significantly reduces hospitalization duration and improves clinical outcomes in cardiac surgery patients [32]. Thus, we propose that TEG-based coagulation monitoring enhances the prediction of perioperative excessive bleeding in patients with ATAAD, while TEG-based risk-stratified transfusion strategies can effectively reduce its incidence and improve clinical outcomes.

Perioperative blood transfusion remains a standard clinical intervention to address perioperative excessive bleeding in patients with ATAAD, particularly due to hemorrhage or hemodilution from CPB [32]. Given the constraints of limited blood transfusion resources, we have developed a TEG-based risk-stratified transfusion strategy to optimize blood product utilization. To our knowledge, evaluations of TEG-based risk-stratified perioperative transfusion strategies in ATAAD patients are scarce.

Therefore, we conducted this prospective, small-sample, single-center, exploratory study. We propose that the kinetic time (K-time) in TEG (OR: 1.848, *p* = 0.034) demonstrates superior sensitivity in reflecting the synergistic contribution of both fibrinogen and platelets during initial clot formation when compared to ACT (OR: 1.008, *p* = 0.048) [33]. Our analysis identified K-time as a significant and independent risk factor for perioperative excessive bleeding in patients with ATAAD. Prolonged K-time is a risk factor for perioperative excessive bleeding in patients with aTAAD, indicating that these patients are more susceptible to perioperative excessive bleeding after surgery. Thus, we propose that combined monitoring of ACT and K-time provides superior assessment of perioperative excessive bleeding in patients with ATAAD. In this study, we implemented a novel risk stratification using ACT and K-time (high-risk: ACT ≥ 140 s and K-time ≥ 1.75 min; low-risk: ACT < 140 s or K-time < 1.75 min). Based on this single-center exploratory study, we established and prospectively validated a risk-stratified transfusion protocol. The ACT/K-time-based risk-stratified transfusion strategy reduced the incidence of perioperative excessive bleeding in patients with aTAAD (9 [34.6%] vs. 1 [4.8%], *p* = 0.033). In our study, the open-label design may have introduced operational bias, as clinicians’ awareness of group assignment could have influenced perioperative management. However, in this study, we used objective outcome measures—chest tube drainage volume and coagulation parameters—to further assess postoperative coagulation recovery, which helped mitigate detection bias to some extent.

In addition, we found that platelet distribution width (PDW) showed a statistically significant difference between the two groups (*p* = 0.027); however, it was not identified as an independent predictor of perioperative excessive bleeding in the multivariate logistic analysis. Previous relevant clinical studies have demonstrated that platelet parameters such as PDW play important predictive roles in the prognosis of patients with conditions such as sepsis [34] and severe burns [35]. We believe this provides some explanation for the observed phenomenon; nevertheless, whether PDW is an independent predictor of perioperative excessive bleeding in patients with aTAAD and whether it can serve as a risk stratification indicator for transfusion therapy warrant further investigation.

We propose that high-risk patients demonstrate significantly worse preoperative coagulation profiles [36]. For high-risk patients, we recommend a targeted transfusion protocol consisting of 8 units of PRBCs, 1000 mL of fresh frozen plasma (FFP), 11 units of cryoprecipitate, and 2 units of platelet concentrates. This transfusion strategy holds the potential to correct preoperative coagulation abnormalities in high-risk patients and reduce the need for blood transfusions due to perioperative excessive bleeding, thereby showing considerable promise for optimizing the utilization of limited blood resources. Pfeiffer’s study [37] demonstrated that adequate hemotherapy effectively restored blood volume while reducing postoperative coagulopathy incidence. For low-risk patients, we propose a more conservative regimen of 5 units of PRBCs, 700 mL of FFP, 8 units of cryoprecipitate, and 1 unit of platelet concentrates (*p* < 0.05). We propose that for low-risk patients, transfusion strategies should focus on meeting clinical needs while minimizing blood product usage, as excessive transfusion does not improve outcomes. Fortunately, our study yielded such encouraging results. The incidence of perioperative excessive bleeding in the retrospective cohort (31.6%) was similar to that in the prospective conventional group (34.6%), suggesting that patient characteristics and surgical procedures remained relatively stable across the two study periods, which, to some extent, strengthens the significance of our findings. Existing studies by Hemli [38] and Sultan [14] further corroborate that excessive perioperative blood transfusion strategies are significantly associated with adverse patient outcomes. Conventional transfusion protocols typically rely on hemoglobin (Hb) and hematocrit (HCT) thresholds or institutional empirical guidelines [10]. Yet, these approaches lack standardization, and outcomes vary widely across centers [39]. Although we observed meaningful results in the above study, the fact that we did not individualize transfusion volumes based on patient body weight or body surface area does, to some extent, limit the generalizability of our findings.

For coronary artery bypass grafting and complex cardiac surgeries, TEG-based risk-stratified transfusion strategies have demonstrated reduced blood product utilization while maintaining therapeutic efficacy and improving patient prognosis [40,41,42]. The TEG-based risk-stratified transfusion strategy holds considerable potential to reduce perioperative consumption of blood products and improve postoperative coagulation function, which is consistent with the findings of our study. Although the TEG-based risk-stratified transfusion strategy in our study achieved notable outcomes in terms of 30-day mortality and perioperative excessive bleeding in patients with aTAAD, we acknowledge that the small sample size and the occurrence of zero mortality events suggest a promising trend toward improved prognosis with this strategy; however, larger-scale studies are warranted to confirm its benefits.

Although the results are promising, this study has several limitations. It was a single-center, exploratory study with a relatively small sample size due to strict inclusion criteria aimed at reducing confounding factors. The TEG-based transfusion strategy may have been influenced by our institutional experience, potentially limiting generalizability. The Youden index was applied to the same dataset used for model development, which may introduce overfitting; therefore, the identified cutoffs require external validation. Given the exploratory nature, we did not adjust for multiple comparisons to avoid increasing type II errors for secondary outcomes. Larger multicenter studies are needed to validate our findings across diverse clinical settings. Moreover, the influence of different circulatory arrest temperatures on perioperative bleeding in aTAAD patients was not detailed in this study and requires future investigation.

## 5. Conclusions

Preoperative TEG parameters, particularly when combined with ACT, can effectively predict perioperative excessive bleeding in patients with aTAAD. Implementing a TEG-based risk-stratified transfusion strategy holds significant potential to improve clinical outcomes, optimize blood product utilization, and reduce costs. This approach provides an evidence-based framework for perioperative blood management in this high-risk population and offers promise for alleviating the strain on blood resource systems.

## Figures and Tables

**Table 1:** Characteristics of Patients In the Retrospective Study Component.

Variable	Normal Perioperative Blood Loss Group (*n* = 39)	Perioperative Excessive Bleeding Group (*n* = 18)	*p* Value
**Demographic**			
Gender, Male, *n* (%)	27 (69.2%)	16 (88.9%)	0.203
Age, (years, median [IQR])	56.00 [44.50, 72.00]	56.50 [49.00, 61.75]	0.77
BMI, (kg/m^2^, median [IQR])	25.59 [21.52, 27.64]	24.07 [22.12, 25.50]	0.291
**Preoperative**			
RBC, (10^12^/L, median [IQR])	4.21 [3.48, 4.74]	4.38 [3.37, 4.57]	0.817
Hb, (g/L, median [IQR])	128.00 [105.00, 141.00]	127.50 [101.00, 142.00]	0.817
PLT, (10^9^/L, median [IQR])	137.00 [98.50, 173.50]	140.00 [100.50, 171.75]	0.777
Platelet volume distribution Width, (fL, median [IQR])	15.20 [12.85, 16.45]	16.50 [15.93, 16.60]	**0.027**
APTT, (s, median [IQR])	28.20 [25.25, 32.85]	28.20 [26.75, 29.75]	0.738
PT, (s, median [IQR])	12.50 [11.80, 14.00]	12.70 [11.83, 14.47]	0.548
INR (median [IQR])	1.09 [1.04, 1.16]	1.10 [1.03, 1.16]	0.918
**Postoperative**			
RBC, (10^12^/L, median [IQR])	3.39 [2.88, 3.64]	2.86 [2.70, 3.32]	0.066
Hb, (g/L, median [IQR])	97.00 [89.00, 108.50]	89.00 [80.25, 102.50]	0.097
PLT, (10^9^/L, median [IQR])	95.00 [81.50, 134.00]	81.00 [70.25, 128.50]	0.229
PT, (s, median [IQR])	14.40 [13.30, 15.55]	14.50 [13.53, 15.90]	0.706
APTT, (s, median [IQR])	33.70 [29.20, 41.65]	38.35 [31.15, 51.25]	0.466
INR (median [IQR])	1.28 [1.17, 1.39]	1.32 [1.20, 1.45]	0.319
**Prognosis**			
Drainage, (mL, median [IQR])	340.00 [250.00, 480.00]	840.00 [812.50, 1075.00]	**<0.001**
Time in hospital, (days, median [IQR])	11.00 [8.00, 28.75]	11.25 [9.25, 22.60]	0.693
Death in 30 days, *n* (%) ^1^	3 (7.7%)	2 (11.1%)	0.999

^1^ Death in 30 days: Death was defined as occurring within 30 days after discharge. Bold ***p*** values indicate statistical significance (*p* < 0.05). In the data, the abbreviations stand for the following: BMI (body mass index), RBC (red blood cell count), Hb (hemoglobin), PLT (platelet count), APTT (activated partial thromboplastin time), PT (prothrombin time), and INR (international normalized ratio).

**Table 2:** Operative Profiles of the Retrospective Study.

Variable	Normal Perioperative Blood Loss Group (*n* = 39)	Perioperative Excessive Bleeding Group (*n* = 18)	*p* Value
**Surgical details**			
Surgery time, (h, median [IQR])	6.25 [5.00, 7.15]	6.50 [6.00, 7.71]	0.232
CPB, (min, median [IQR])	176.00 [147.00, 205.00]	200.00 [186.50, 216.50]	0.099
DHCA, (min, median [IQR])	24.00 [19.00, 27.00]	28.00 [18.25, 33.75]	0.449
Bleeding amount, (mL, median [IQR])	1500.00 [1200.00, 1950.00]	2100.00 [1525.00, 2875.00]	**0.012**
Packed red blood cells, *n* (%)			**0.025**
0–4 IU	16 (41.0%)	2 (11.1%)	
4.5–10 IU	19 (48.7%)	10 (55.6%)	
>10 IU	4 (10.3%)	6 (33.3%)	
Fresh frozen plasma, *n* (%)			0.339
0–600 mL	17 (43.6%)	5 (27.8%)	
600–800 mL	13 (33.3%)	5 (27.8%)	
800–1000 mL	6 (15.4%)	4 (22.2%)	
>1000 mL	3 (7.7%)	4 (22.2%)	
Cryoprecipitate, *n* (%)			0.286
0–10 IU	25 (64.1%)	12 (66.7%)	
10–15 IU	10 (25.6%)	2 (11.1%)	
>15 IU	4 (10.3%)	4 (22.2%)	
Platelet concentrates, *n* (%)			**0.022**
0–1 IU	29 (74.4%)	7 (38.9%)	
>1 IU	10 (25.6%)	11 (61.1%)	

Bold ***p*** values indicate statistical significance (*p* < 0.05). In the data, the abbreviations stand for the following: CPB (cardiopulmonary bypass), DHCA (deep hypothermic circulatory arrest).

**Table 3:** Characteristics of Thromboelastography Results in the Retrospective Study.

Variable	Normal Perioperative Blood Loss Group (*n* = 39)	Perioperative Excessive Bleeding Group (*n* = 18)	*p* Value
**Preoperative**			
ACT, (s, median [IQR])	128.00 [117.00, 140.00]	140.00 [128.00, 173.00]	0.085
R, (min, median [IQR])	0.80 [0.75, 0.95]	0.95 [0.80, 1.28]	0.099
K, (min, median [IQR])	1.80 [1.35, 2.50]	2.90 [2.15, 3.60]	**0.001**
α Angle, (deg, median [IQR])	71.90 [66.10, 75.90]	64.30 [55.97, 75.00]	0.078
MA, (mm, median [IQR])	61.50 [56.20, 66.85]	59.45 [52.90, 64.85]	0.315
A, (mm, median [IQR])	62.60 [57.00, 68.25]	60.05 [53.78, 65.98]	0.349
G, (Kd/sc, median [IQR])	8.00 [6.45, 10.05]	7.35 [5.60, 9.25]	0.315
**Postoperative**			
R, (min, median [IQR])	8.00 [7.15, 9.75]	8.65 [7.12, 11.23]	0.764
K, (min, median [IQR])	2.00 [1.45, 2.60]	2.35 [2.02, 2.58]	0.179
α Angle, (deg, median [IQR])	63.00 [55.85, 69.25]	61.45 [52.52, 63.72]	0.415
MA, (mm, median [IQR])	58.20 [53.85, 63.65]	50.65 [48.42, 55.20]	**0.001**
A, (mm, median [IQR])	58.50 [53.80, 64.05]	54.45 [51.62, 58.77]	0.067
CI, (median [IQR])	−2.60 [−4.20, −0.75]	−2.80 [−4.97, −1.83]	0.376
G, (Kd/sc, median [IQR])	7.00 [5.80, 9.15]	6.50 [5.62, 8.83]	0.823

Bold ***p*** values indicate statistical significance (*p* < 0.05). In the data, the abbreviations stand for the following: ACT (activated clotting time), R (reaction time), K (kinetic time), MA (maximum amplitude), CI (coagulation index), A (amplitude), G (shear elastic modulus).

**Table 4:** Univariate Logistic Regression Analysis of the TEG-related results.

Variable	OR	0.025	0.975	B	Wald	*p* Value
**Preoperative**						
ACT	1.008	1.002	1.019	0.008	3.919	**0.048**
R	2.146	1.151	5.664	0.764	3.862	**0.049**
K	1.848	1.108	3.476	0.614	4.489	**0.034**
α Angle	0.934	0.871	0.995	−0.068	4.249	**0.039**
MA	0.97	0.91	1.029	−0.031	1.001	0.317
A	0.973	0.913	1.034	−0.027	0.764	0.382
G	0.911	0.75	1.068	−0.093	1.104	0.293
TMA	1.156	1	1.365	0.145	3.444	0.063
**Postoperative**						
R	0.964	0.793	1.173	−0.036	0.137	0.711
K	1.235	0.636	2.389	0.211	0.409	0.523
α Angle	0.986	0.937	1.038	−0.014	0.31	0.578
MA	0.864	0.774	0.944	−0.146	8.663	**0.003**
A	0.929	0.851	1.004	−0.073	3.155	0.076
CI	0.937	0.759	1.152	−0.065	0.388	0.533
G	1	1	1.001	0	1.033	0.309

Bold ***p*** values indicate statistical significance (*p* < 0.05). In the data, the abbreviations stand for the following: ACT (activated clotting time), R (reaction time), K (kinetic time), MA (maximum amplitude), CI (coagulation index), A (amplitude), G (shear elastic modulus), TMA (time to maximum amplitude).

**Table 5:** Preoperative TEG-based Risk-stratified Transfusion Strategy.

High-risk Group (ACT ≥ 140 AND K-time ≥ 1.75)	Packed red blood cells: 8 IU; Fresh frozen plasma: 1000 mL; Cryoprecipitate: 11 IU; Platelet concentrates: 2 IU *
Low-risk Group (ACT < 140 OR K-time < 1.75)	Packed red blood cells: 5 IU; Fresh frozen plasma: 700 mL; Cryoprecipitate: 8 IU; Platelet concentrates: 1 IU *

* Recommended transfusion volumes were as follows: packed red blood cells (8 ± 0.5 IU or 5 ± 0.5 IU), fresh frozen plasma (1000 ± 100 mL or 700 ± 100 mL), cryoprecipitate (11 ± 1 IU or 8 ± 1 IU), and platelet concentrates (2 ± 0.5 IU or 1 ± 0.5 IU), all administered in accordance with the established transfusion protocol. The predefined transfusion volumes were based on an estimated average body weight of 70 kg for male patients and 60 kg for female patients, with modest adjustments permitted according to actual body weight. This transfusion strategy was developed based on a literature review, including established TEG-based risk-stratified transfusion protocols in cardiac surgery, combined with clinical experience from our institution.

**Table 6:** Comparison of Prognostic Outcomes Between TEG-based Risk-stratified Transfusion and Empirical Transfusion.

Variable	The Empirical Transfusion Practice Group (*n* = 21)	The TEG-Based Risk-Stratified Transfusion Protocol Group (*n* = 26)	*p* Value
**Prognosis**			
Drainage, (mL, median [IQR])	400.00 [350.00, 547.50]	320.00 [170.00, 470.00]	**0.046**
Time in hospital, (days, median [IQR])	13.00 [8.50, 18.00]	17.00 [14.00, 21.00]	0.153
Fee, (yuan, median [IQR])	159,202.04 [113,734.32, 167,401.24]	153,134.02 [109,873.76, 175,499.35]	0.732
Transfusion fee, (yuan, median [IQR])	6787.41 [5741.74, 8807.26]	5608.29 [4602.42, 7495.24]	**0.029**
Perioperative excessive bleeding, *n*, (%)	9 (34.6%)	1 (4.8%)	**0.033**
Death in 30 days, *n*, (%)	7 (33.3%)	0	**0.030**

Bold ***p*** values indicate statistical significance (*p* < 0.05).

## Data Availability

The raw data supporting the conclusions of this article will be made available by the authors on request.

## Supplementary Materials

The following supporting information can be downloaded at: https://www.mdpi.com/article/10.3390/jcm15093446/s1, Supplementary Figure S1: Results of the Correlation Analysis; Supplementary Figure S2: Results of the ROC Curve Analysis for Each Predictor; Supplementary Figure S3: ROC and calibration curves; Supplementary Table S1: Characteristics of Patients In the Retrospective Study Component; Supplementary Table S2: Univariate Logistic Regression Analysis; Supplementary Table S3: Multivariable Analysis Results; Supplementary Table S4: Characteristics of Patients In the Prospective Study Component.

## Abbreviations

The following abbreviations are used in this manuscript:

INR	international normalized ratio
BMI	body mass index
WBC	white blood cell count
RBC	red blood cell
eGFR	estimated glomerular filtration rate
PT	prothrombin time
APTT	activated partial thromboplastin time
CPB	cardiopulmonary bypass
DHCA	deep hypothermic circulatory arrest
ACT	activated clotting time
Hb	hemoglobin
HCT	hematocrit
PLT	platelets
ATAAD	acute type A aortic dissection
CABG	coronary artery bypass grafting
pRBC	packed red blood cell
TEG	thromboelastography
R-time	reaction time
K-time	kinetic time
MA	maximum amplitude
LY30	lysis at 30 min
CI	coagulation index
EPL	estimated percent lysis
ROC	Receiver Operating Characteristic
FFP	fresh frozen plasma
A	amplitude
G	shear elastic modulus
OR	Odds Ratio
