# Supplementary material for: Thromboelastography-Based Risk-Stratified Transfusion Strategy in Acute Stanford Type A Aortic Dissection: A Predictive Model and Prospective Validation
Source: J Clin Med. 2026 Apr 30;15(9):3446. doi: 10.3390/jcm15093446 (PMC13163563; doi:10.3390/jcm15093446)
Supplement: Supplementary file 1 [file jcm-15-03446-s001.zip › jcm-4130211-supplementary.pdf]

Supplementary

1.1 Surgical procedures

All patients underwent emergency/urgent surgical repair for aTAAD under general anesthesia. CPB was established via femoral and/or axillary arterial cannulation and bicaval or right atrial cannulation after systemic heparinization(administer heparin at 300 IU per kilogram body weight to achieve systemic anticoagulation, targeting an ACT > 480 seconds).

Intraoperative cerebral protection utilized primarily unilateral or bilateral selective antegrade cerebral perfusion. Unilateral selective antegrade cerebral perfusion was performed under deep hypothermic circulatory arrest at 22°C, whereas bilateral selective antegrade cerebral perfusion was performed under moderate hypothermic circulatory arrest at 27–28°C. Aortic repair strategies were individualized based on entry tear location, extent of dissection, and surgical judgment. Root management involved either replacement or reconstruction, while arch procedures included hemiarch replacement, total arch replacement(often with frozen elephant trunk), island anastomosis, or stent-based techniques (triple-branched or fenestrated). Concomitant procedures such as CABG or valve surgery were performed as indicated[21].

Following weaning from cardiopulmonary bypass, heparin anticoagulation was systematically reversed through administration of protamine sulfate at a fixed 1:1 ratio relative to the initial heparin dose. Transfusion therapy was initiated immediately after weaning from cardiopulmonary bypass to correct coagulation function and hemoglobin levels. Chest closure is performed only after confirming hemodynamic stability and satisfactory surgical hemostasis.

Supplementary **Table S1**: Characteristics of Patients In the Retrospective Study Component

Variable	Normal perioperative blood loss group (*n*=39)	Perioperative excessive bleeding group(*n*=18)	P value
**Preoperative comorbidities**			
Chest pain , *n* , (%)	34 (87.2%)	17 (94.4%)	0.714
Back pain , *n* , (%)	22 (56.4%)	11 (61.1%)	0.964
Transient ischemic attack , *n* , (%)	4 (10.3%)	3 (16.7%)	0.802
Consciousness , *n* , (%)	1 (2.6%)	1 (5.6%)	0.999
Hypertension , *n* , (%)	31 (79.5%)	16 (88.9%)	0.622
Diabetes , *n* , (%)	0	1 (5.6%)	0.689
Coronary artery disease history , *n* , (%)	1 (2.6%)	3 (16.7%)	0.168
Smoke , *n* , (%)	7 (17.9%)	4 (22.2%)	0.985
Alcohol , *n* , (%)	7 (17.9%)	3 (16.7%)	0.999
Hypotension , *n* , (%)	0	2 (11.1%)	0.179
Limb ischemia , *n* , (%)	0	3 (16.7%)	**0.048**
Myocardial ischemia , *n* , (%)	2 (5.1%)	3 (16.7%)	0.354
**Preoperative**			
WBC , (10^9/L , median [IQR])	11.80 [9.55, 14.00]	9.70 [7.60, 12.47]	0.154
Neutrophil percentage , (% , median [IQR])	86.20 [80.25, 88.70]	75.95 [69.00, 88.75]	0.054
Monocyte percentage , (% , median [IQR])	6.80 [4.70, 7.90]	5.70 [4.53, 7.37]	0.268
Basophil percentage , (% , median [IQR])	0.10 [0.10, 0.20]	0.15 [0.10, 0.48]	0.259
Eosnophils percentage , (% , median [IQR])	0.10 [0.00, 0.10]	0.20 [0.03, 0.50]	**0.039**
Lymphocyte percentage , (% , median [IQR])	6.60 [4.70, 10.50]	9.20 [3.45, 17.12]	0.706
HCT , (L/L , median [IQR])	38.00 [31.45, 41.65]	38.90 [30.62, 42.25]	0.857
URBC , (n/μL , median [IQR])	123.00 [16.50, 776.50]	43.00 [20.00, 580.25]	0.797
UWBC , (n/μL , median [IQR])	12.00 [8.00, 21.42]	13.00 [5.75, 22.60]	0.999
ALT , (U/L , median [IQR])	27.00 [18.35, 44.35]	27.20 [18.75, 39.75]	0.952
AST , (U/L , median [IQR])	38.00 [28.00, 76.50]	34.95 [27.50, 55.50]	0.757
LDH , (U/L , median [IQR])	437.00 [363.00, 584.00]	441.50 [336.75, 667.50]	0.784
Total bilirubin , (μmol/L , median [IQR])	15.50 [12.25, 20.40]	16.10 [12.90, 22.42]	0.643
Direct bilirubin , (μmol/L , median [IQR])	10.80 [6.35, 13.95]	9.00 [6.73, 15.07]	0.823
Total protein , (g/L , median [IQR])	64.20 [58.30, 70.00]	61.30 [51.85, 67.05]	0.319
Albumin , (g/L , median [IQR])	38.50 [35.70, 41.75]	37.50 [31.27, 39.50]	0.186
HDL , (mmol/L , median [IQR])	0.90 [0.73, 1.10]	0.86 [0.68, 1.00]	0.264
LDL , (mmol/L , median [IQR])	1.29 [0.96, 1.69]	1.06 [0.76, 1.35]	0.070

Total cholesterol , (mmol/L , median [IQR])	2.85 [2.46, 3.27]	2.46 [1.98, 2.89]	0.092
eGFR , (mL/min/1.73m^2 , median [IQR])	82.39 [52.05, 128.25]	63.90 [23.70, 82.39]	**0.028**
Creatinine , (μmol/L , median [IQR])	66.00 [56.10, 109.75]	95.80 [70.55, 121.40]	0.055
Fibrinogen , (g/L , median [IQR])	2.60 [1.90, 3.10]	2.10 [1.80, 3.15]	0.405
D-dimmer , (mg/L , median [IQR])	8.18 [3.26, 16.50]	10.43 [5.96, 21.46]	0.279
CKMB , (ng/mL , median [IQR])	16.00 [7.00, 37.57]	10.50 [7.25, 33.93]	0.869
**Surgical details**			
Aortic cross-clamping , (min , median [IQR])	122.00 [104.00, 155.50]	150.50 [120.75, 169.50]	0.122
DHCA , (min , median [IQR])	24.00 [19.00, 27.00]	28.00 [18.25, 33.75]	0.449
Total amount of blood transfusion , (mL , median [IQR])	2390.00 [1942.50, 2961.25]	3255.00 [2082.50, 4215.00]	0.118
**Postoperative**			
WBC , (10^9/L , median [IQR])	9.20 [7.40, 12.05]	9.10 [7.93, 10.17]	0.699
Neutrophil percentage , (% , median [IQR])	6.70 [4.35, 9.25]	5.65 [3.82, 8.50]	0.220
Monocyte percentage , (% , median [IQR])	0.00 [0.00, 0.10]	0.10 [0.00, 0.25]	0.585
Basophil percentage , (% , median [IQR])	7.90 [6.35, 10.25]	7.65 [6.60, 8.73]	0.391
Eosnophils percentage , (% , median [IQR])	0.60 [0.50, 0.75]	0.40 [0.20, 0.80]	0.094
Lymphocyte percentage , (% , median [IQR])	0.01 [0.00, 0.01]	0.01 [0.00, 0.01]	0.244
ALT , (U/L , median [IQR])	36.80 [27.70, 62.80]	42.10 [31.50, 162.65]	0.257
AST , (U/L , median [IQR])	19.90 [14.10, 30.20]	43.95 [14.88, 143.28]	**0.048**
Total bilirubin , (μmol/L , median [IQR])	25.90 [17.20, 37.00]	22.85 [16.83, 29.62]	0.341
Direct bilirubin , (μmol/L , median [IQR])	12.70 [7.70, 18.45]	10.45 [7.55, 14.30]	0.509
LDH , (U/L , median [IQR])	440.00 [365.00, 551.50]	465.00 [362.25, 733.25]	0.349
Total cholesterol , (mmol/L , median [IQR])	2.66 [2.31, 3.08]	2.38 [2.19, 2.81]	0.201
Total protein , (g/L , median [IQR])	54.10 [51.80, 58.30]	53.10 [51.55, 57.68]	0.680
Albumin , (g/L , median [IQR])	36.30 [34.25, 38.55]	35.60 [34.38, 38.12]	0.536
Creatinine , (μmol/L , median [IQR])	78.00 [60.00, 140.00]	102.50 [72.50, 214.75]	0.069
HDL , (mmol/L , median [IQR])	0.66 [0.52, 0.82]	0.55 [0.40, 0.66]	0.087
LDL , (mmol/L , median [IQR])	1.12 [0.90, 1.46]	1.15 [0.78, 1.30]	0.415
Fibrinogen , (g/L , median [IQR])	2.50 [2.20, 3.40]	2.50 [2.00, 3.15]	0.362
D-dimmer , (mg/L , median [IQR])	13.10 [7.90, 13.10]	13.10 [12.83, 17.04]	0.173

Data are presented as median [interquartile range] or n (%). Group comparisons were performed using the Mann-Whitney U test (continuous variables) or Fisher's exact test (categorical variables). A P value < 0.05 was considered statistically significant. Abbreviations: WBC, white blood cell count; ALT, alanine

aminotransferase; AST, aspartate aminotransferase; HDL, high-density lipoprotein; LDL, low-density lipoprotein; eGFR, estimated glomerular filtration rate; DHCA, deep hypothermic circulatory arrest; HCT, hematocrit; UWBC, urinary white blood cell count; URBC, urinary red blood cell count; LDH, lactate dehydrogenase; CKMB, creatine ki

Supplementary **Table S2**: Univariate Logistic Regression Analysis

Variable	OR	2.50%	97.50%	B	Wald	P value
**Demographic**						
Gender	0.281	0.040	1.207	-1.269	2.356	0.125
Age	0.989	0.950	1.029	-0.011	0.277	0.599
BMI	0.930	0.795	1.072	-0.072	0.936	0.333
**Preoperative comorbidities**						
Chest pain	2.500	0.364	49.912	0.916	0.652	0.419
Back pain	1.214	0.392	3.924	0.194	0.112	0.738
Transient ischemic attack	1.750	0.313	8.906	0.560	0.462	0.497
Consciousness	2.235	0.085	58.804	0.804	0.310	0.577
Hypertension	2.065	0.451	14.760	0.725	0.730	0.393
Coronary artery disease history	7.600	0.894	160.521	2.028	2.884	0.089
Smoke	1.306	0.302	5.081	0.267	0.144	0.704
Alcohol	0.914	0.178	3.810	-0.090	0.014	0.906
Myocardial ischemia	3.700	0.560	30.306	1.308	1.846	0.174
**Preoperative**						
WBC	0.898	0.756	1.046	-0.108	1.739	0.187
Neutrophil percentage	0.967	0.928	0.994	-0.033	4.206	**0.040**
Monocyte percentage	0.904	0.737	1.099	-0.101	1.017	0.313
Basophil percentage	1.175	0.966	1.533	0.161	2.248	0.134
Eosnophils percentage	2.966	1.013	11.615	1.087	3.353	0.067
Lymphocyte percentage	1.056	0.970	1.154	0.055	1.596	0.206
RBC	0.855	0.450	1.606	-0.157	0.242	0.623
Hb	1.000	0.978	1.022	0.000	0.000	0.983
HCT	0.999	0.925	1.080	-0.001	0.001	0.971
PLT	0.998	0.988	1.008	-0.002	0.193	0.660
Platelet volume distribution Width	1.643	1.119	2.770	0.497	4.863	**0.027**
URBC	1.000	0.999	1.000	0.000	0.527	0.468
UWBC	1.000	0.978	1.018	0.000	0.000	0.999
ALT	1.002	0.999	1.005	0.002	1.325	0.250
AST	1.001	1.000	1.004	0.001	1.049	0.306
LDH	1.000	0.999	1.001	0.000	0.015	0.902
Total bilirubin	1.004	0.946	1.060	0.004	0.020	0.888
Direct bilirubin	0.983	0.904	1.052	-0.017	0.206	0.650
Total protein	0.967	0.914	1.019	-0.034	1.563	0.211
Albumin	0.938	0.855	1.023	-0.064	2.014	0.156
HDL	0.205	0.019	1.665	-1.587	1.982	0.159
LDL	0.341	0.086	0.976	-1.075	2.999	0.083
Total cholesterol	0.468	0.178	0.995	-0.760	2.991	0.084

eGFR	0.983	0.967	0.997	-0.017	5.064	**0.024**
Creatinine	1.001	0.996	1.006	0.001	0.183	0.669
APTT	1.038	0.998	1.144	0.037	0.985	0.321
PT	1.120	1.008	1.377	0.113	1.892	0.169
Fibrinogen	0.816	0.487	1.269	-0.203	0.724	0.395
INR	2.661	0.216	48.808	0.979	0.632	0.426
D-dimmer	1.020	0.976	1.067	0.020	0.822	0.365
**Surgical details**						
Surgery time	1.281	0.895	1.888	0.247	1.768	0.184
CPB	1.008	0.996	1.023	0.008	1.589	0.207
Aortic cross-clamping	1.008	0.994	1.023	0.008	1.214	0.270
DHCA	1.021	0.992	1.063	0.021	1.514	0.219
Bleeding amount	1.001	1.000	1.001	0.001	4.009	**0.045**
Total amount of blood transfusion	1.000	1.000	1.001	0.000	2.188	0.139
Packed red blood cells	3.416	1.407	9.656	1.228	6.486	**0.011**
Fresh frozen plasma	1.627	0.945	2.895	0.487	2.992	0.084
Cryoprecipitate	1.189	0.544	2.512	0.173	0.204	0.651
Platelet concentrates	2.900	0.904	9.599	1.065	3.178	0.075
**Postoperative**						
WBC	0.907	0.721	1.117	-0.098	0.793	0.373
Neutrophil percentage	0.910	0.753	1.080	-0.094	1.075	0.300
Monocyte percentage	0.645	0.044	1.704	-0.439	0.326	0.568
Basophil percentage	0.843	0.670	1.030	-0.171	2.543	0.111
Eosnophils percentage	0.170	0.021	1.028	-1.773	3.297	0.069
RBC	0.347	0.108	0.935	-1.060	3.778	0.052
Hb	0.965	0.925	1.000	-0.036	3.304	0.069
PLT	0.993	0.978	1.007	-0.007	0.851	0.356
ALT	1.004	1.000	1.011	0.004	2.669	0.102
AST	1.005	1.000	1.012	0.005	3.072	0.080
Total bilirubin	0.996	0.957	1.030	-0.004	0.047	0.828
Direct bilirubin	1.001	0.940	1.059	0.001	0.003	0.959
LDH	1.002	1.000	1.005	0.002	2.441	0.118
Total cholesterol	0.635	0.209	1.760	-0.454	0.718	0.397
Total protein	0.980	0.883	1.082	-0.020	0.161	0.688
Albumin	0.927	0.766	1.117	-0.076	0.639	0.424
Creatinine	1.003	0.999	1.008	0.003	1.771	0.183
HDL	0.105	0.006	1.256	-2.250	2.750	0.097
LDL	0.849	0.219	2.942	-0.164	0.064	0.800
PT	1.009	0.827	1.239	0.009	0.008	0.928
APTT	1.007	0.973	1.043	0.007	0.181	0.670

INR	5.440	0.565	72.093	1.694	2.027	0.155
Fibrinogen	0.763	0.417	1.285	-0.271	0.929	0.335
D-dimmer	1.054	0.974	1.147	0.053	1.674	0.196

Univariate logistic regression analysis with perioperative excessive bleeding as the dependent variable. OR, odds ratio; 0.025 and 0.975 represent the 95% confidence interval of the OR; B, regression coefficient; Wald, Wald χ^2 value. Bold P values indicate statistical significance ($P < 0.05$). Abbreviations: INR, international normalized ratio; BMI, body mass index; RBC, red blood cell count; Hb, hemoglobin; PT, prothrombin time; APTT, activated partial thromboplastin time; CPB, cardiopulmonary bypass; ACT, activated clotting time; R, reaction time; MA, maximum amplitude; CI, comprehensive coagulation index. Other abbreviations are as defined in Supplementary Table S1.

Supplementary **Table S3**: Multivariable Analysis Results

Var	OR	0.025	0.975	B	Wald	P value
ALL	0.0147	0.0000	1734906907	-4.220	0.1096	0.7406
Preoperative ACT	1.0656	0.7557	1.5191	0.0636	0.1348	0.7135
Preoperative R	0.0051	0.0000	5.04352E+11	-5.287	0.1065	0.7442
Preoperative K	1.2225	0.5197	3.2076	0.2009	0.2122	0.6451
Preoperative α Angle	1.0047	0.8684	1.1681	0.0047	0.004	0.9496
Preoperative Neutrophil percentage	0.9657	0.9184	1.0011	-0.035	2.8816	0.0896
Preoperative eGFR	0.9826	0.9623	0.9996	-0.018	3.4022	0.0651
Packed red blood cells	3.7826	1.2217	14.6929	1.3304	4.6198	**0.0316**

Multivariable logistic regression analysis using a stepwise selection method (or the variable selection approach described). Variables with $P < 0.10$ in univariate analysis and clinically important variables were included. The final model included: preoperative ACT, preoperative R, preoperative K, preoperative α angle, preoperative neutrophil percentage, preoperative eGFR, and packed red blood cells. VIF (variance inflation factor) values were all < 5, indicating no significant collinearity. Bold P value (packed red blood cells) is statistically significant. Abbreviations: ACT, activated clotting time; R, reaction time; K, coagulation time; α Angle, alpha angle; eGFR, estimated glomerular filtration rate. VIF：Preoperative ACT: 1.23, Preoperative R: 2.45, Preoperative K: 1.65; Preoperative α Angle: 1.89, Preoperative Neutrophil percentage: 1.67, Preoperative eGFR: 1.12, Packed red blood cells: 1.05.

Supplementary **Table S4**: Characteristics of Patients In the Prospective Study Component

Variable	The empirical transfusion practice group(n=21)	The TEG-based risk-stratified transfusion protocol group(n=26)	P value
**Demographic**			
Gender, Male, *n* (%)	22 (84.6%)	18 (85.7%)	0.999
Age, (years , median [IQR])	54.50 [42.50, 59.75]	58.00 [51.00, 63.00]	0.380
BMI, (kg/m^2 , median [IQR])	26.92 [24.68, 29.05]	24.91 [23.67, 27.68]	0.167
**Preoperative**			
ACT , (sec , median [IQR])	136.00 [121.00, 152.00]	121.00 [113.00, 136.00]	0.136
R , (min , median [IQR])	0.90 [0.80, 1.10]	0.80 [0.70, 0.90]	0.151
K , (min , median [IQR])	1.40 [1.20, 1.90]	1.30 [0.90, 1.80]	0.177
α Angle , (deg , median [IQR])	74.90 [70.85, 76.97]	76.40 [71.80, 79.80]	0.104
MA , (mm , median [IQR])	60.60 [44.32, 67.78]	65.20 [59.40, 71.10]	**0.035**
A , (mm , median [IQR])	62.45 [54.80, 68.77]	66.20 [59.40, 71.90]	0.146
G , (Kd/sc , median [IQR])	8.00 [3.98, 10.52]	9.35 [7.30, 12.33]	0.054
TMA , (min , median [IQR])	16.20 [11.96, 17.78]	16.30 [14.80, 17.80]	0.422
WBC , (10^9/L , median [IQR])	6.80 [4.30, 9.78]	6.50 [5.10, 8.20]	0.773
Neutrophil percentage , (% , median [IQR])	63.20 [52.72, 87.72]	64.00 [59.00, 82.30]	0.789
Monocyte percentage , (% , median [IQR])	25.05 [7.43, 37.22]	25.40 [12.00, 33.40]	0.692
Basophil percentage , (% , median [IQR])	6.50 [5.25, 7.83]	6.60 [5.50, 6.90]	0.700
Eosnophils percentage , (% , median [IQR])	1.60 [0.15, 3.70]	1.50 [0.20, 2.90]	0.923
Lymphocyte percentage , (% , median [IQR])	0.35 [0.10, 0.80]	0.50 [0.10, 0.60]	0.888
RBC , (10^12/L , median [IQR])	3.95 [3.67, 4.62]	4.23 [3.93, 4.47]	0.435
Hb , (g/L , median [IQR])	121.00 [110.25, 139.75]	128.00 [112.00, 135.00]	0.600
HCT , (L/L , median [IQR])	36.45 [34.07, 42.08]	39.10 [35.50, 41.80]	0.454
PLT , (10^9/L , median [IQR])	129.50 [99.00, 181.25]	157.00 [144.00, 171.00]	0.174
ALT , (U/L , median [IQR])	18.20 [11.90, 37.05]	15.90 [11.40, 24.10]	0.822
AST , (U/L , median [IQR])	21.40 [14.85, 36.70]	22.20 [17.10, 43.00]	0.563
LDH , (U/L , median [IQR])	200.50 [174.00, 336.25]	216.00 [184.00, 395.00]	0.467
Total bilirubin , (μmol/L , median [IQR])	11.05 [7.72, 15.35]	13.00 [9.00, 21.90]	0.266
Direct bilirubin , (μmol/L , median [IQR])	2.85 [1.95, 5.03]	2.90 [2.40, 7.10]	0.460
Total protein , (g/L , median [IQR])	63.50 [60.23, 66.38]	66.70 [62.00, 69.70]	0.149
Albumin , (g/L , median [IQR])	38.10 [35.23, 39.30]	39.50 [36.60, 41.90]	0.257
HDL , (mmol/L , median [IQR])	1.08 [0.84, 1.33]	1.08 [0.87, 1.36]	0.789
LDL , (mmol/L , median [IQR])	2.01 [1.64, 2.37]	2.23 [1.54, 2.79]	0.357

Total cholesterol , (mmol/L , median [IQR])	3.78 [3.36, 4.03]	3.99 [3.39, 4.55]	0.231
eGFR , (mL/min/1.73m^2 , median [IQR])	106.60 [84.40, 125.65]	112.80 [93.30, 117.20]	0.653
Creatinine , (μmol/L , median [IQR])	66.50 [59.00, 80.00]	67.00 [62.00, 75.00]	0.999
APTT , (s , median [IQR])	28.30 [26.88, 31.22]	27.90 [27.30, 30.40]	0.822
PT , (s , median [IQR])	12.00 [11.03, 12.95]	11.60 [11.20, 12.60]	0.661
Fibrinogen , (g/L , median [IQR])	2.40 [2.20, 2.98]	3.10 [2.10, 3.70]	0.234
INR (median [IQR])	1.06 [0.96, 1.15]	1.02 [0.98, 1.11]	0.661
**Surgical details**			
Surgery time , (hours , median [IQR])	6.10 [6.00, 7.00]	6.00 [6.00, 7.10]	0.605
CPB , (min , median [IQR])	184.00 [171.50, 212.75]	206.00 [188.00, 233.00]	0.140
Aortic cross-clamping , (min , median [IQR])	135.00 [118.50, 153.00]	165.00 [144.00, 196.00]	**0.012**
Leukoreduced Red Blood Cells , (mL , median [IQR])	1250.00 [1000.00, 2525.00]	1500.00 [1200.00, 2700.00]	0.575
Blood Plasma , (mL , median [IQR])	850.00 [718.75, 1631.25]	775.00 [750.00, 1400.00]	0.629
Cryoprecipitation , (mL , median [IQR])	11.25 [7.81, 17.50]	9.00 [8.00, 16.00]	0.614
Platelet , (mL , median [IQR])	2.00 [1.00, 2.00]	1.00 [1.00, 2.00]	0.313
DHCA , (min , median [IQR])	24.50 [22.00, 32.50]	34.00 [24.00, 42.00]	0.059
**Postoperative**			
R , (min , median [IQR])	0.80 [0.70, 0.90]	0.90 [0.80, 0.90]	0.073
K , (min , median [IQR])	2.20 [1.70, 2.40]	2.20 [1.40, 2.40]	0.796
α Angle , (deg , median [IQR])	70.75 [69.88, 74.90]	70.70 [68.90, 74.70]	0.940
MA , (mm , median [IQR])	52.70 [51.70, 59.38]	52.70 [52.70, 58.40]	0.863
A , (mm , median [IQR])	56.90 [52.92, 60.18]	56.90 [53.40, 61.10]	0.880
A30 , (mm , median [IQR])	-52.50 [-59.55, -52.10]	-52.60 [-58.80, -52.50]	0.504
G , (Kd/sc , median [IQR])	6.39 [5.36, 7.31]	6.39 [5.56, 7.01]	0.966
WBC , (10^9/L , median [IQR])	9.85 [7.47, 12.30]	10.70 [8.20, 12.90]	0.500
Neutrophil percentage , (% , median [IQR])	86.65 [78.85, 88.52]	86.10 [83.40, 91.90]	0.289
Monocyte percentage , (% , median [IQR])	7.50 [5.03, 12.43]	5.40 [4.10, 9.30]	0.248
Basophil percentage , (% , median [IQR])	6.05 [5.20, 7.75]	6.00 [4.00, 7.30]	0.309
Eosnophils percentage , (% , median [IQR])	0.10 [0.00, 0.38]	0.10 [0.10, 0.30]	0.759
Lymphocyte percentage , (% , median [IQR])	0.10 [0.10, 0.20]	0.20 [0.10, 0.30]	0.202
RBC , (10^12/L , median [IQR])	3.72 [3.27, 4.08]	3.47 [3.11, 3.80]	0.149
Hb , (g/L , median [IQR])	111.50 [98.25, 126.00]	104.00 [92.00, 113.00]	0.192
HCT , (L/L , median [IQR])	34.45 [29.30, 37.70]	33.50 [27.90, 34.90]	0.195

PLT , (10^9/L , median [IQR])	112.00 [89.75, 158.25]	106.00 [77.00, 140.00]	0.991
ALT , (U/L , median [IQR])	18.20 [11.90, 37.05]	15.90 [11.40, 24.10]	0.822
AST , (U/L , median [IQR])	21.40 [14.85, 36.70]	22.20 [17.10, 43.00]	0.563
Total bilirubin , (μmol/L , median [IQR])	11.05 [7.72, 15.35]	13.00 [9.00, 21.90]	0.266
Direct bilirubin , (μmol/L , median [IQR])	2.85 [1.95, 5.03]	2.90 [2.40, 7.10]	0.460
LDH , (U/L , median [IQR])	354.00 [301.00, 396.50]	402.00 [336.00, 457.00]	0.097
Total cholesterol , (mmol/L , median [IQR])	3.10 [2.83, 3.37]	2.78 [2.48, 3.58]	0.128
Total protein , (g/L , median [IQR])	58.90 [56.52, 60.55]	57.60 [55.50, 61.60]	0.422
Albumin , (g/L , median [IQR])	35.70 [33.45, 37.05]	34.90 [33.60, 38.20]	0.653
Creatinine , (μmol/L , median [IQR])	62.50 [54.50, 78.80]	65.00 [54.00, 78.80]	0.622
eGFR,mL/min/1.73m^2(median [IQR])	23.40 [21.60, 24.00]	22.80 [18.90, 24.30]	0.493
HDL , (mmol/L , median [IQR])	0.80 [0.66, 1.02]	0.73 [0.63, 1.00]	0.340
LDL , (mmol/L , median [IQR])	1.60 [1.30, 1.88]	1.33 [0.91, 1.88]	0.256
PT , (s , median [IQR])	13.00 [12.20, 14.00]	13.90 [13.00, 15.20]	**0.035**
APTT , (s , median [IQR])	35.50 [29.52, 44.60]	33.80 [31.30, 35.50]	0.940
INR (median [IQR])	1.15 [1.07, 1.25]	1.23 [1.15, 1.36]	**0.034**
Fibrinogen , (g/L , median [IQR])	2.55 [2.15, 2.98]	2.20 [1.90, 3.00]	0.520
	**Prognosis**		
Drainage , (mL , median [IQR])	400.00 [350.00, 547.50]	320.00 [170.00, 470.00]	**0.046**
Time in hospital , (days , median [IQR])	13.00 [8.50, 18.00]	17.00 [14.00, 21.00]	0.153
Fee , (yuan , median [IQR])	159202.04 [113734.32, 167401.24]	153134.02 [109873.76, 175499.35]	0.732
Transfusion fee, (yuan, median [IQR])	6787.41 [5741.74, 8807.26]	5608.29 [4602.42, 7495.24]	**0.029**
Perioperative excessive bleeding , *n* , (%)	9 (34.6%)	1 (4.8%)	**0.033**
Death in 30 days , *n* , (%)	7 (33.3%)	0	**0.030**

Data are presented as median [interquartile range] or n (%). Group comparisons were performed using the Mann-Whitney U test (continuous variables) or Fisher's exact test (categorical variables). A P value < 0.05 was considered statistically significant. Abbreviations: INR, international normalized ratio; BMI, body mass index; WBC, white blood cell count; RBC, red blood cell count; Hb, hemoglobin; ALT, alanine aminotransferase; AST, aspartate aminotransferase; HDL, high-density lipoprotein; LDL, low-density lipoprotein; eGFR, estimated glomerular filtration rate; PT, prothrombin time; APTT, activated partial thromboplastin time; CPB, cardiopulmonary bypass; DHCA, deep hypothermic circulatory arrest; ACT, activated clotting time; R, reaction time; MA, maximum amplitude; CI, comprehensive coagulation index; HCT, hematocrit; PLT, platelets; LDH, lactate dehydrogenase; CSA-AKI, cardiac surgery-associated acute kidney injury. Death within 30 days was defined as death occurring within 30 days after discharge. Prognosis: The TEG-guided group showed significantly less postoperative drainage, lower transfusion costs, and lower incidences of perioperative excessive bleeding and 30-day mortality.

Supplementary **Figure S1**: Results of the Correlation Analysis

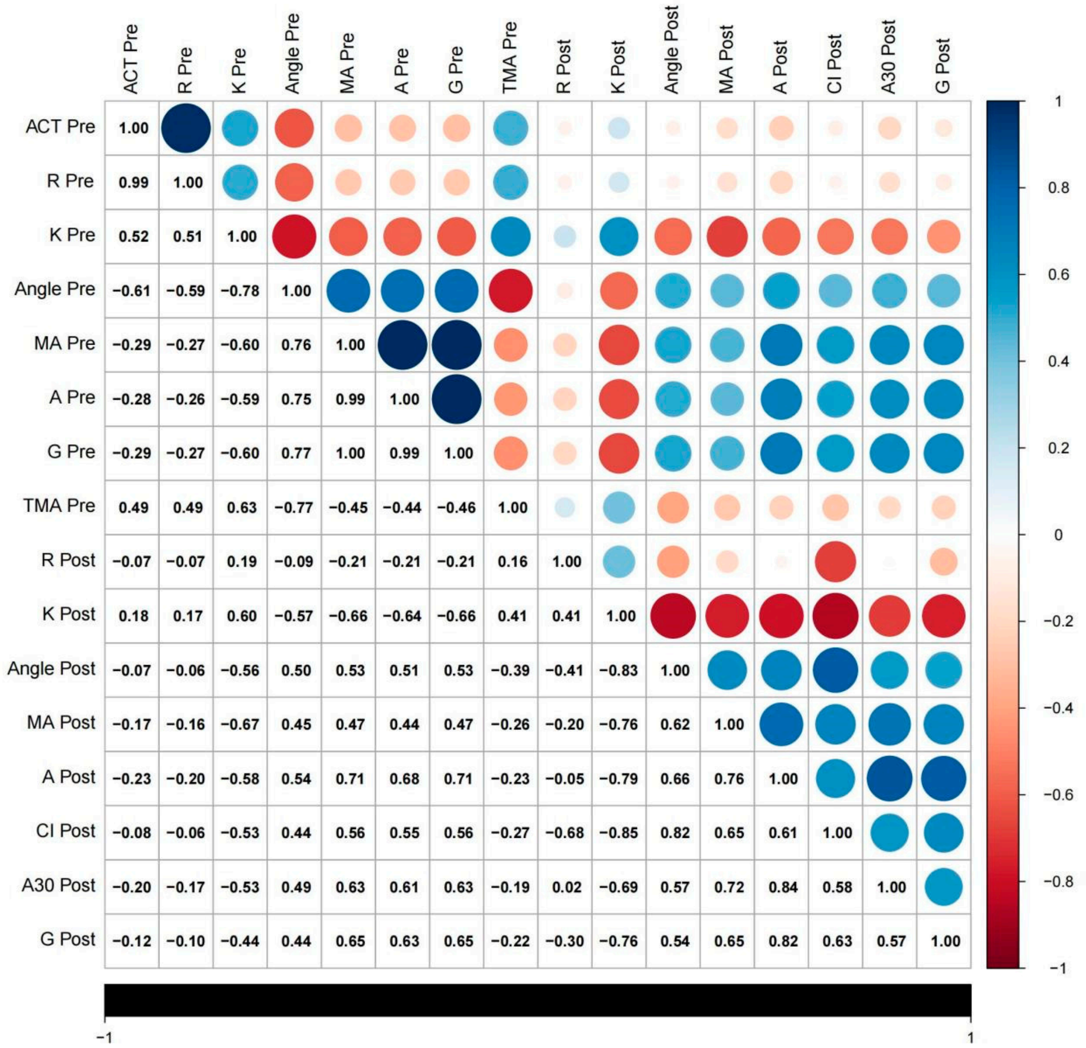

Correlation matrix of preoperative and postoperative thromboelastography (TEG) parameters. The colour intensity and numbers represent the correlation coefficients. Abbreviations: Pre/Post ACT, activated clotting time; Pre/Post R, reaction time; Pre/Post K, coagulation time; Pre/Post Angle, α angle; Pre/Post MA, maximum amplitude; Pre A, amplitude at a certain time point; Pre G, shear elastic modulus; Pre TMA, time to maximum amplitude; Post CI, coagulation index; Post A30, amplitude at 30 minutes after MA.

Supplementary **Figure S2**: Results of the ROC Curve Analysis for Each Predictor

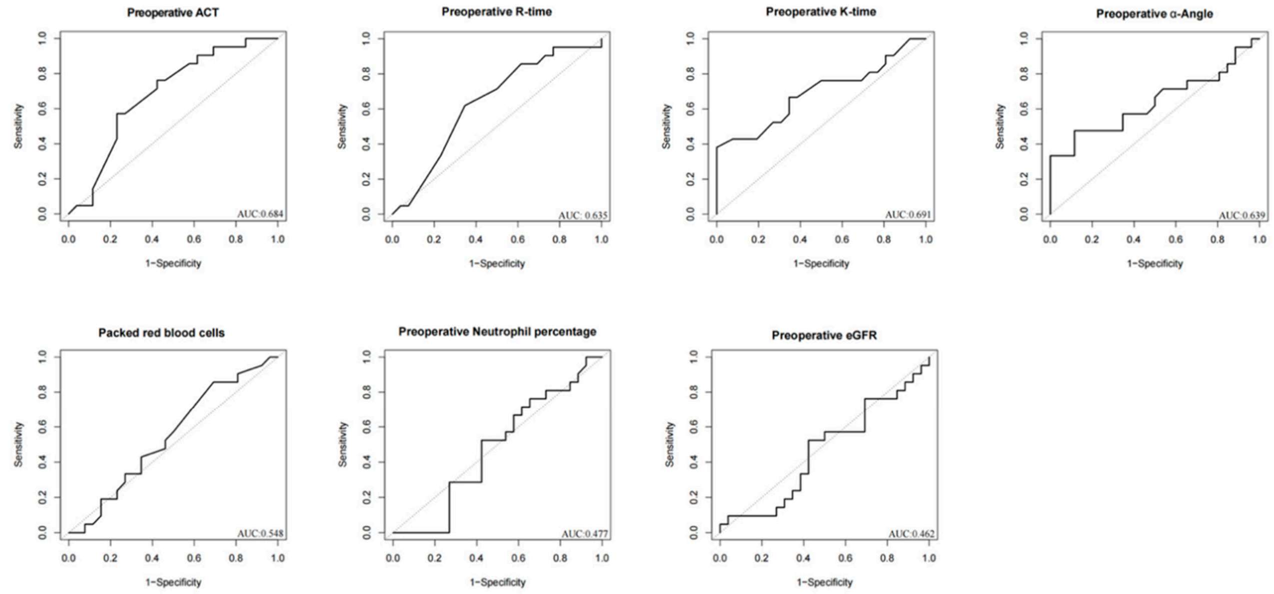

Receiver operating characteristic (ROC) curves for the prediction of perioperative excessive bleeding. Individual predictive variables (preoperative ACT, preoperative R-time, preoperative K-time, preoperative α-angle, packed red blood cells, preoperative neutrophil percentage, and preoperative eGFR) are listed below the graph.

Supplementary **Figure S3**: ROC and calibration curves

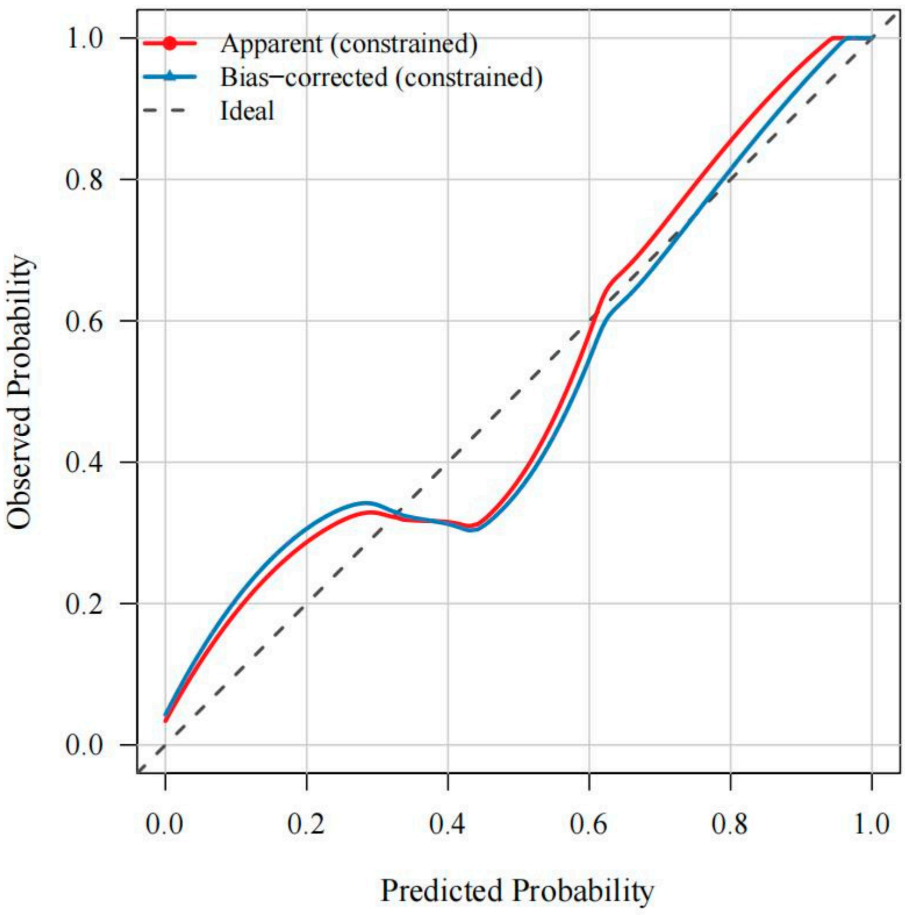

Calibration curve of the multivariable logistic regression model for perioperative excessive bleeding. The x-axis shows the predicted probability, and the y-axis shows the observed probability. The apparent curve (non-corrected), bias-corrected curve (using bootstrapping), and the ideal reference line (45° diagonal) are displayed. Good calibration is indicated by close agreement between the bias-corrected curve and the ideal line.

Reference

21. Xue Y, Pan J, Cao H, Fan F, Luo X, Ge M, Chen Y, Wang D, Zhou Q. Different aortic arch surgery methods for type A aor-tic dissection: clinical outcomes and follow-up results. Interact Cardiovasc Thorac Surg. 2020, 31, 254-262. https://doi.org/10.1093/icvts/ivaa095.
